# Temporal Expectations Guide Dynamic Prioritization in Visual Working Memory through Attenuated α Oscillations

**DOI:** 10.1523/JNEUROSCI.2272-16.2016

**Published:** 2017-01-11

**Authors:** Freek van Ede, Marcel Niklaus, Anna C. Nobre

**Affiliations:** ^1^Oxford Centre for Human Brain Activity, Department of Psychiatry, and; ^2^Department of Experimental Psychology, University of Oxford, OX3 7JX, Oxford, United Kingdom, and; ^3^Department of Psychology, University of Zurich, 8050 Zurich, Switzerland

**Keywords:** α oscillations, attention, neuronal oscillations, temporal attention, working memory

## Abstract

Although working memory is generally considered a highly dynamic mnemonic store, popular laboratory tasks used to understand its psychological and neural mechanisms (such as change detection and continuous reproduction) often remain relatively “static,” involving the retention of a set number of items throughout a shared delay interval. In the current study, we investigated visual working memory in a more dynamic setting, and assessed the following: (1) whether internally guided temporal expectations can dynamically and reversibly prioritize individual mnemonic items at specific times at which they are deemed most relevant; and (2) the neural substrates that support such dynamic prioritization. Participants encoded two differently colored oriented bars into visual working memory to retrieve the orientation of one bar with a precision judgment when subsequently probed. To test for the flexible temporal control to access and retrieve remembered items, we manipulated the probability for each of the two bars to be probed over time, and recorded EEG in healthy human volunteers. Temporal expectations had a profound influence on working memory performance, leading to faster access times as well as more accurate orientation reproductions for items that were probed at expected times. Furthermore, this dynamic prioritization was associated with the temporally specific attenuation of contralateral α (8–14 Hz) oscillations that, moreover, predicted working memory access times on a trial-by-trial basis. We conclude that attentional prioritization in working memory can be dynamically steered by internally guided temporal expectations, and is supported by the attenuation of α oscillations in task-relevant sensory brain areas.

**SIGNIFICANCE STATEMENT** In dynamic, everyday-like, environments, flexible goal-directed behavior requires that mental representations that are kept in an active (working memory) store are dynamic, too. We investigated working memory in a more dynamic setting than is conventional, and demonstrate that expectations about when mnemonic items are most relevant can dynamically and reversibly prioritize these items in time. Moreover, we uncover a neural substrate of such dynamic prioritization in contralateral visual brain areas and show that this substrate predicts working memory retrieval times on a trial-by-trial basis. This places the experimental study of working memory, and its neuronal underpinnings, in a more dynamic and ecologically valid context, and provides new insights into the neural implementation of attentional prioritization within working memory.

## Introduction

Working memory pertains to the fundamental cognitive ability to retain and manipulate currently relevant information in an active store, in service of ongoing task demands (Baddeley, 1992; D'Esposito and Postle, 2015). Accordingly, working memory is an essential component within everyday tasks, such as monitoring surrounding traffic when driving a car, or comprehending speech. To support adaptive behavior in such dynamic tasks, it is evident that working memory representations, too, must be highly dynamic. Despite this consideration, however, commonly used laboratory tasks of working memory (such as change detection and continuous reproduction) require the retention of a set number of items throughout a shared retention interval. Here, we investigated working memory in a more dynamic setting, asking whether and how internally guided temporal expectations can lead to the dynamic prioritization of different items in working memory at different times. We also investigated the neural substrates of such dynamic prioritization using EEG.

Over the past decade, it has become well established that the fate of perceptual working memory representations can continue to be influenced after encoding. For example, attentional cues presented during the retention interval (retro-cues) facilitate subsequent responses about the cued item (Griffin and Nobre, 2003; Landman et al., 2003; Pertzov et al., 2013; Souza and Oberauer, 2016). These studies have thus demonstrated that spatial and/or object-based attentional biases continue to operate during working memory and have led to the view that, among several items in working memory, a subset of the items (usually one), can be in a “prioritized state” (Cowan, 1988; Oberauer, 2002) (i.e., in the “focus of attention”).

From the perceptual domain, there is ample evidence that temporal expectations provide another potent source of attentional biasing (Coull and Nobre, 1998; Ghose and Maunsell, 2002; Nobre et al., 2007). Based on this work, we hypothesized that temporal expectations also continue to operate during working memory, by facilitating the accessibility and the accuracy of mnemonic representations at those times at which they are deemed most relevant. We were interested in testing, therefore, whether prioritization of an item for access in working memory could be controlled flexibly through prediction about when its retrieval was required. We were additionally interested in the neural substrates supporting such flexible item prioritization.

In the perceptual domain, the attenuation of 8–14 Hz α oscillations is a prominent index of the allocation of attention (Foxe et al., 1998; Worden et al., 2000; Thut et al., 2006; Klimesch et al., 2007), which is associated with the engagement of the underlying neuronal populations (Jensen and Mazaheri, 2010; Haegens et al., 2011). More recently, attenuated α oscillations over task-relevant sensory areas have also been implicated in the retention of perceptual representations in working memory (Jokisch and Jensen, 2007; Sauseng et al., 2009; Spitzer and Blankenburg, 2012). Furthermore, several recent studies have suggested that spatially specific decreases in α oscillations are triggered by orienting attention to an item's spatial location in working memory (e.g., Myers et al., 2014; Poch et al., 2014; Wallis et al., 2015; Mok et al., 2016).

It has not yet been established, however, whether such attentional α modulations during working memory also (1) support temporal attention; in accordance with observations in the perceptual domain (Rohenkohl and Nobre, 2011; van Ede et al., 2011), and (2) predict mnemonic performance on a trial-by-trial basis. Moreover, prior studies could not rule out lateralized retro-cue processing and/or probe anticipation. To avoid these potential confounds in the present study, we manipulated spatiotemporal expectations through learned associations with the item colors (instead of asymmetrical retro-cues) and presented probes centrally (instead of at the peripheral location of the item during encoding).

We thus hypothesize that temporal expectations can dynamically guide which item in visual working memory is currently prioritized, and that this will be reflected in measures of working memory performance, as well as in spatially and temporally specific modulations of posterior α oscillations that are relevant for working memory performance.

## Materials and Methods

Twenty-four healthy human volunteers (13 male, mean age: 24 years, range: 19–34 years) participated in the study. Based on previous EEG studies, we aimed at a sample size of ∼25 but stopped at 24 for counterbalancing purposes (i.e., to ensure an equal number of participants with each color-delay mapping). Data from all participants were retained for analysis. The experiment was approved by the Central University Research Ethics Committee of the University of Oxford. Participants provided written informed consent before participation and were paid £10/h.

### 

#### 

##### Experimental design and procedure.

**Figure 1.**
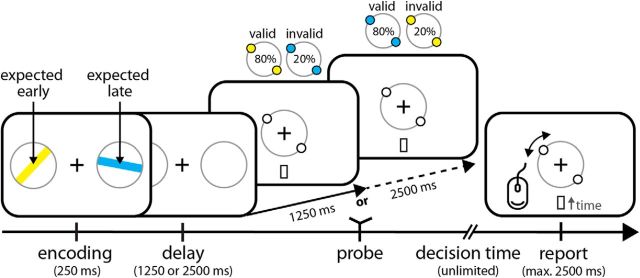
Task. During encoding, two randomly oriented bars were presented for 250 ms. Bars were always positioned to the left and right of fixation, and one bar was always yellow, whereas the other was blue (color-side mapping was randomly determined for each trial). After a delay of either 1250 or 2500 ms, either of the items was probed by a central probe stimulus. Color of the handles of the central probe (as illustrated above the early and late probe displays in the schematic) represents the to-be-reproduced item. The key manipulation was that the probability of being probed about either the blue or the yellow item was varied over time (with color-interval mappings being counterbalanced across participants). Schematic represents a case in which yellow is expected early and blue late, as indicated by the percentages above the early and late probe displays. Participants reproduced the orientation of the probed item using the mouse. After the probe display appeared, participants were given unlimited time to retrieve the item from working memory and decide what to report. However, once they started to move the mouse, they were given limited time (2500 ms) to complete their report. Elapsed time was displayed under the probe.

Participants performed a visual working memory task (see Fig. 1) that required the short-term retention of two oriented peripheral bars: one on the left and one on the right of a screen. One was always yellow (RGB colors: 255,241,0) and the other was always blue (RGB colors: 0,173,238). Bar orientations were randomly and independently drawn for each item and trial. In each trial, participants were probed about only one item, at one of two possible delays (1250/2500 ms) after encoding (which lasted 250 ms). The key manipulation was that, if probed early, the item of one color was most likely to be probed, whereas, if probed late, the other colored item was most likely. The passing of the early interval (in late probe trials) was never explicitly indicated to participants. Color-delay mappings were 80% valid and were counterbalanced across participants (while kept constant within a given participant). Figure 1 illustrates the case for which the yellow item is expected early, and the blue item late. Participants were informed about the color-delay mapping at the start of the experiment and practiced one block of 40 trials to acquaint themselves with the relevant intervals.

At a viewing distance of 90 cm, bars had a diameter of 6.4 degrees visual angle and were centered at 6.4 degrees visual angle to the left and right of fixation. Color-side mappings were randomly varied on a trial-by-trial basis. To promote a visual strategy for working memory retention, we presented the items inside “placeholders” (i.e., see Fig. 1, gray circles) that were kept on the screen throughout the retention interval. Critically, however, these placeholders contained no information regarding the relevant mnemonic variable, which was orientation.

Working memory was probed using a continuous reproduction task. A probe was presented in the center of the screen that consisted of a gray circle (with the same diameter as the oriented bars) with two “handles” (see Fig. 1). Handles were placed on opposite sides to indicate the probe's orientation. Participants' task was to reproduce the orientation of the probed item by aligning these handles to the remembered orientation, using the mouse. The color of the handles (yellow/blue) indicated which item was to be reported. One of the handles had a slightly thicker edge, marking the current position of the mouse's cursor. The starting orientation was drawn randomly for each trial.

Following the appearance of the probe screen, participants were given unlimited time to decide what to report. However, once they started to move the mouse, they were given only limited time (2500 ms) to complete their orientation reproduction. Elapsed time was displayed under the probe (see Fig. 1). Before time would run out, participants could click the mouse to verify their response and continue with the task. Immediately after a response, feedback was provided: if the reported orientation was within 15 degrees of the target orientation, the fixation cross turned green for 250 ms (with brighter greens for more precise reports); otherwise, it turned red. Intertrial intervals were drawn randomly between 750 and 1000 ms.

Participants completed 15 blocks of 40 trials each (totaling 600 trials). Between blocks, participants performed a short eye-tracker calibration session and were presented with a (task-free) visual localizer that was included to assist the EEG analysis. The visual localizer consisted of 40 randomly drawn oriented bars that were each presented for 250 ms, either to the left or right of fixation. Localizer stimuli matched the task stimuli in terms of color, location, and size. Interstimulus intervals were randomly drawn between 300 and 400 ms.

Our EEG analysis aimed to capitalize on the fact that the items that were expected to be probed early and late were presented in different hemifields. This analysis thus assumes some degree of “retinotopic preservation” of the mnemonic items during working memory retention (as in Kuo et al., 2009). To this end, two elements of our experimental design aimed to promote a retinotopically specific retention strategy. First, the visual items were presented inside placeholders, and these placeholders were left on the screen throughout the retention interval (assisting participants to remember the relevant orientation information at the encoded locations). Second, the probe's location was identical for both items. Whereas under certain circumstances participants may “shift” the mnemonic item to the to-be-probed location soon after encoding (Zaksas et al., 2001; Pasternak and Zaksas, 2003), in our experiment this would likely lead to interference between both items because the to-be-probed location is the same for both items. Therefore, keeping the items at their encoded locations during retention may help reduce interference between them.

##### Analysis of behavioral data.

We focused on two behavioral variables of interest. First, we calculated the average error of the reproduction report by taking the absolute angular difference between the target orientation and the reported orientation, and averaged this value across all trials within each condition. Second, we calculated the average decision time for each condition, which was defined as the time between the onset of the probe screen and the start of the reproduction report. All trials for which the decision time was >5 SDs above the mean decision time were excluded from the analysis. For both dependent variables, we subjected the participant-specific averages to a repeated-measures ANOVA with the factors “expected time” and “probed time.” Complementary to this, we also assigned conditions as “valid” and “invalid” and compared these classes using paired-sample *t* tests.

##### EEG acquisition and analysis.

We acquired EEG using Synamps amplifiers and Neuroscan data acquisition software (Compumedics). We used a custom 42 channel setup with the following subset of electrodes of the international 10–10 system: FPz, AFz, AF3/4, AF7/8, Fz, F1/2, F3/4, FCz, FC1/2 FC5/6, Cz, C3/4, T7/8, CP1/2, CP5/6, Pz, P1/2, P3/4, P5/6, P7/8, POz, PO3/4, PO7/8, Oz, O1/2, thus providing most dense coverage over posterior sites of interest (see also Fig. 3*a*). The left mastoid was used as the active reference, and we included a right mastoid measurement to derive an average-mastoid reference offline. The ground was placed on the left upper arm. During acquisition, data were low-pass filtered by an anti-aliasing filter (250 Hz cutoff), digitized at 1000 Hz, and stored for offline analysis.

EEG data were analyzed in MATLAB using FieldTrip (Oostenveld et al., 2011) (RRID: SCR_004849). During data preprocessing, we removed line noise using a discrete Fourier Transform filter, cut out our epochs of interest, and removed excessively noisy epochs based on visual inspection of the signal's variance across trials and channels. This was done once for variance calculated on the broadband signal and, subsequently, once for variance calculated on the 8–30 Hz bandpass-filtered signal (to detect artifacts specific to the frequency range of interest). Artifact rejection was performed on all trials, without knowledge of the conditions to which trials belonged.

##### Localizer-based channel selection.

We first analyzed our visual localizer data to find the electrodes that were most susceptible to visual processing of the left and right stimulus locations. We did this separately for each participant. To this end, we contrasted left and right visual localizer stimuli with regard to (8–14 Hz) α power in the 150–400 ms poststimulus window and expressed this as a relative change (i.e., [left − right]/[left + right] × 100). The subset of the electrodes (usually four, but see below) showing the most negative (positive) contrast values were then selected to represent the right (left) visual areas. We aimed to select four electrodes on either side, but depending on the specific pattern that we observed, we sometimes picked three of five. Critically, this selection was made before looking at the task data of interest. To confirm that this approach converged on meaningful channel selections, we depict the proportion of participants for which a given channel was selected in Figure 3*a*.

##### Time-frequency analysis.

We used a short-time Fourier transform to estimate time- and frequency-resolved estimates of oscillatory power. We estimated power for frequencies between 5 and 30 Hz (in steps of 1 Hz), and used a 400 ms sliding time window that was advanced in steps of 80 ms. We obtained time-frequency maps for each of the selected channels and collapsed across the channels belonging to the same (left/right) visual channel clusters. For each channel cluster, we then contrasted trials in which the item contralateral to the cluster was expected early versus trials in which this item was expected late (i.e., [early − late]/[early + late] × 100). Because of our design, this is equivalent to contrasting trials in which the item expected early was contralateral versus ipsilateral to the channel cluster. We calculated this contrast of interested separately for both channel clusters, and collapsed their outcomes. To avoid contamination of visual processing of early probes, we only included time windows in which this probe had not yet occurred. As a result, the expectation-related time-frequency maps (as depicted in Figs. 3*b*, 4*b*) were based on all trials in the early interval, but based on only the late-probe trials in the late interval.

##### Trialwise correlations with performance.

For our trialwise correlation analysis, we centered time-frequency maps at probe onset and evaluated correlations with working memory performance separately for the channel clusters that were contralateral and ipsilateral to the side of the relevant (i.e., expected and probed) mnemonic item at encoding. We only included valid trials because only in these trials are contralateral and ipsilateral loci defined relative to both preprobe and postprobe time windows. Provided that oscillatory power and behavioral performance are each likely to covary with time-on-task, we calculated partial correlations in which we included trial numbers to factor out this potential contribution.

##### Statistical approach.

The independent visual localizer allowed us to focus our analysis of the task data on the most relevant visual channel clusters while avoiding selection bias. Using these channel clusters, we were able to reduce our analysis to several targeted time-frequency maps (as described above). Statistical testing of the EEG data focused exclusively on these time-frequency maps. To this end, we used a cluster-based permutation approach (Maris and Oostenveld, 2007), which circumvents the multiple-comparison problem by evaluating the full time-frequency space under a single permutation distribution of the largest cluster. However, to better appreciate the plausibility (see van Ede and Maris, 2016) of the patterns behind these significant clusters, we also plotted spatial maps and bar graphs associated with these clusters. However, these were presented solely for descriptive purposes and were (deliberately) not subjected to further statistical tests.

##### Eye-tracking acquisition and analysis.

Gaze was monitored using an EyeLink 1000 eye-tracker (SR Research). Data were sampled at 1000 Hz and stored for offline analysis. Offline, we synchronized the gaze data with the EEG data on the basis of event triggers that were sent to both the EEG and the eye-tracker acquisition devices. A custom eye-tracker calibration session was included after every block in which participants were asked to track a green square that was presented at the same eccentricities of the visual stimuli that were used in the experiment. On the basis of these data, we determined the eye-tracker values that were associated with these locations, which allowed us to quantify the degree to which participants' gaze was biased toward these locations during the experiment.

## Results

Participants performed a visual working memory task that required the short-term retention of two oriented bars, one yellow and one blue (Fig. 1). After either a short (1250 ms) or a long (2500 ms) delay, participants were probed about one of the items and were asked to reproduce its orientation. From the outset of a trial (i.e., at encoding), early and late probes were always equiprobable, making both items equally relevant. The key manipulation was that we varied which item was most likely to be probed over time. If probed early, the item of one specific color was four times more likely to be probed than the item of the other color. However, if this interval passed, and participants would thus be probed late, the other colored item became four times more likely. Tracking of the passage of time thus enabled participants to know which item was most likely to be probed at any given time. This is illustrated in Figure 1 for a case in which the yellow item is more likely early, and the blue item late (assignment of which color was likely to be probed early vs late was counterbalanced across participants).

### Temporal expectations influence the accuracy and accessibility of working memory representations

**Figure 2.**
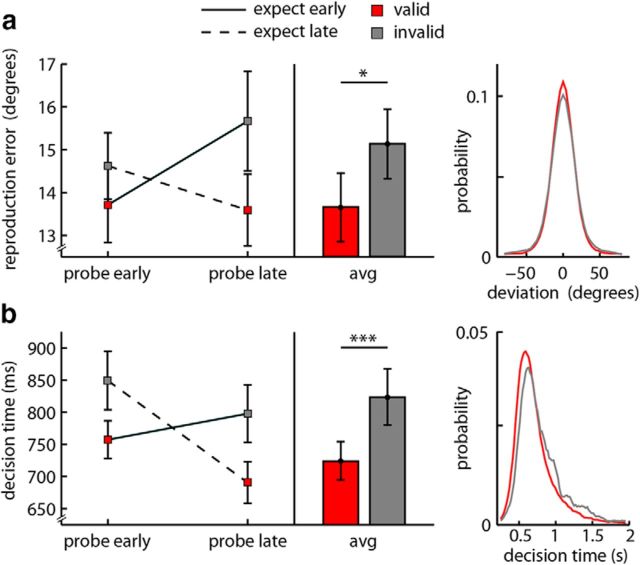
Behavioral results. ***a***, Mean reproduction errors (i.e., [abs(reported orientation − target orientation)]) in degrees, as a function of when an item was probed (early/late) and when it was expected to be probed (early/late). Bar graph represents performance for items probed at expected times (valid, red) or unexpected times (invalid, gray). Error bars indicate SEM, calculated across participants. Rightmost panel, Distributions of response deviations (relative to target orientation) for valid and invalid trials. ***b***, Same conventions as in ***a***, except for the dependent variable: decision time.

We first investigated the influence of temporal expectations on the accuracy of working memory representations. To this end, we evaluated the average reproduction error (i.e., the absolute deviation from the target orientation), for which lower values indicate better performance. As depicted in Figure 2*a*, when probed early, errors were smaller for items that were also expected early; conversely, when probed late, errors were smaller for items that were also expected late. Simply put, errors were smaller for items probed at expected times. This was confirmed by a significant interaction between the factors “expected time” and “probed time” (*F*_(1,23)_ = 6.39; *p* = 0.019, η_*p*_^2^ = 0.22), or, equivalently, a significant benefit for temporally valid compared with invalid trials (*t*_(23)_ = −2.52; *p* = 0.019; *d* = −0.52). When considering early and late probe trials separately, however, these validity effects did not survive statistical significance testing (early: *t*_(23)_ = −1.09; *p* = 0.287; *d* = −0.22; late: *t*_(23)_ = −1.91; *p* = 0.069; *d* = −0.39). Average error distributions in Figure 2*a* confirm the subtle nature of the influence of temporal expectations on working memory accuracy, with similar distributions for valid and invalid trials. At the same time, these distributions argue against the possibility that unexpected items were simply dropped from working memory.

In addition to the accuracy of the probed items, we were also interested in the accessibility of their representations. For this, we focused on the time between probe onset and the start of the reproduction report as an index of “decision time” (i.e., how long it takes to access the item before deciding what to report). To increase the usefulness of this measure, participants had unlimited time to decide what to report, but, once they started to move the mouse, only limited time for reproduction.

The influence of temporal expectations on decision time is depicted in Figure 2*b* and reveals a similar, except much more robust, crossover interaction as described for response accuracy above. Participants were faster to decide what to report when items were probed at expected times. This was again confirmed by a highly significant interaction between the factors “expected time” and “probed time” (*F*_(1,23)_ = 22.27; *p* = 9.482 × 10^−5^, η_*p*_^2^ = 0.49), or, equivalently, a highly significant benefit for valid compared with invalid trials (*t*_(23)_ = −4.71; *p* = 9.590 × 10^−5^; *d* = −0.96). Furthermore, for decision time, this validity benefit was significant for both early and late probes (early: *t*_(23)_ = −3.97; *p* = 6.06 × 10^−4^; *d* = −0.81; late: *t*_(23)_ = −3.25; *p* = 0.004; *d* = −0.66). Most prominently, when an item was unexpectedly probed early (which was expected to be probed late), participants required on average ∼850 ms to decide what to report, whereas when the same item was (expectedly) probed late, decisions were ∼150 ms faster (Fig. 2*b*, dashed line). This suggests prominent “recovery” of the item's accessibility when it became more relevant later on in the trial. The temporal validity effect is further visible from the average decision time distributions for valid and invalid trials, as depicted in Figure 2*b*.

### Internally guided reprioritization in visual working memory is associated with spatially and temporally specific modulations of posterior α oscillations

We next investigated the electrophysiological substrates that may support the dynamic prioritization of working memory representations by internally guided temporal expectations. Because yellow and blue items were always positioned on the left and on the right side of the screen (with the color-side mapping being randomly determined on a trial-by-trial basis), we were able to focus our analysis on α power relative to the side of the currently prioritized item (which, due to our design, always involved opposite sides between the early and the late intervals). We investigated this across a time range that spanned both the short and the long retention intervals, but only included in the analysis time intervals in which the probe had not yet occurred (see Materials and Methods).

**Figure 3.**
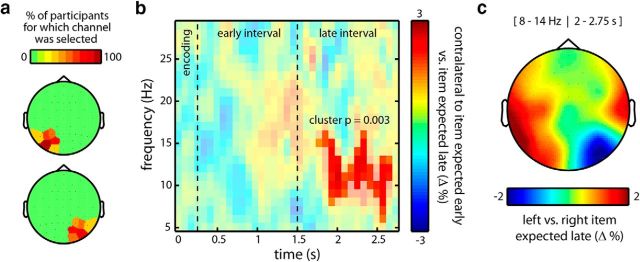
Posterior α modulation during dynamic prioritization in working memory. ***a***, Channel selections for the left and right visual areas, as derived from an independent visual localizer (see Materials and Methods). Color coding represents the percentage of participants for which a given channel was selected to be part of the left (top) or right (bottom) posterior channel clusters. ***b***, Time-frequency plot of the normalized difference in power contralateral to the item expected early versus the item expected late (i.e., [(early − late)/(early + late)] × 100). Only data segments were included in which the probe had not yet occurred (see Materials and Methods). The transparency mask highlights the significant time-frequency cluster (see Materials and Methods). ***c***, Topography of the difference in α power in the late interval, for late expected items that were presented on the left versus on the right at encoding.

To increase the sensitivity of this analysis, we used a visual localizer to select the relevant left and right posterior channels on an individual participant basis (see Materials and Methods). Figure 3*a* depicts the proportion of participants for which a given channel was selected as being part of the left (top) or right (bottom) channel cluster (with channels P07 and PO8 showing the highest proportions). Zooming in on the data from these channel clusters, Figure 3*b* depicts the difference in power between trials in which the item contralateral to the cluster was expected early versus when the item contralateral to the cluster was expected late (note that, due to our design, this is equivalent to [contralateral vs ipsilateral to the item expected early] or [ipsilateral vs contralateral to the item expected late]). Evaluating this contrast across the full time-frequency space (see Materials and Methods) revealed a significant cluster (cluster *p* = 0.003) that peaked between 8 and 14 Hz (i.e., in the α band) and that was sustained throughout most of the late interval (as highlighted in Fig. 3*b*). Because we contrasted contralateral activity for items expected early minus items expected late, positive (red) values imply higher α power contralateral to the item expected early or, equivalently, lower α power contralateral to the item expected late.

The direction of this modulation also becomes evident when considering its topographical distribution (Fig. 3*c*), quantified as the difference between items expected to be probed late that were presented on the left versus on the right at encoding. Indeed, when the item that was expected to be probed late was the left item, there was less α power in the late interval over the right (contralateral) posterior sites (blue in the depicted contrast). Conversely, if this item was presented on the right, there was less α power in this late interval over the left posterior sites (red in the depicted contrast). This topography additionally confirms that this modulation is largely restricted to the same posterior sites that were also found in our independent visual localizer (compare Fig. 3*c* with Fig. 3*a*).

**Figure 4.**
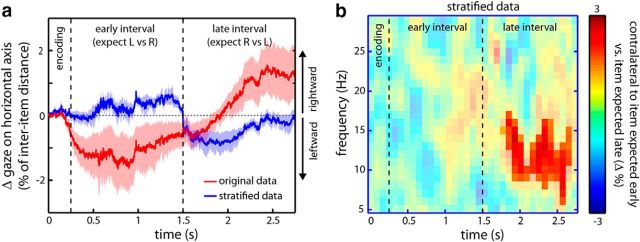
Gaze during dynamic prioritization in working memory and its independence of the α modulation. ***a***, Gaze on the horizontal axis, expressed as a percentage of the interitem distance (as calibrated using an eye-tracker localizer; see Materials and Methods). Data are expressed as the difference in gaze between trials in which the item expected early was on the left versus on the right (which is equivalent to right vs left in the late interval). Red curve indicates the original data and reveals a slight bias of gaze in the direction of the side of the expected item at encoding. Shading represents ± 1 SEM, calculated across participants. Critically, after we removed trials to the point where this gaze bias was reversed (blue curve; see Materials and Methods), the α modulation in Figure 3*b* remained virtually identical, as depicted in ***b***.

We next assessed whether this lateralized α modulation may be accounted for by differences in gaze between trials in which the prioritized items were on the left versus on the right during encoding. Figure 4*a* (red line) depicts the difference in gaze for trials in which the item expected early was the left item versus trials in which the item expected early was the right item (which is equivalent to right vs left in the late interval). These data did reveal a slight (<2%) bias of gaze toward the side of the item that was expected to be probed (<0.13 degree visual angle). Moreover, this gaze bias closely tracked our spatiotemporal expectation manipulation (showing a reversal after the first interval passed and the opposite item became more probable). Critically, however, when we selectively removed trials to the point where, for every participant, average gaze in each interval was away from the side of the expected item (Fig. 4*a*, blue line, stratified data; on average, we had to remove 7.58 ± 1.72% of trials), the observed α modulation remained virtually identical (Fig. 4*b*; compare with Fig. 3*b*). Thus, whereas gaze patterns may also dynamically track the item that is currently prioritized in visual working memory (despite the fact that the probe was anticipated centrally), differences in gaze do not account for the observed α lateralization.

In summary, despite not observing a clear neuronal signature of the focus on attention in the early interval (we return to possible reasons for this in the Discussion), soon after this interval passes, these data reveal attenuated α power contralateral to the item that is brought back (“recovered”) into the focus of attention. As shown above, this lateralized modulation cannot be explained by differential gaze patterns. It can also not be due to probe anticipation because the probe is always presented centrally. Together with the fact that the transition from the early to the late interval is never explicitly cued, it must therefore be attributed to dynamic and temporally precise internally guided processes during the working memory retention period.

### α states associated with prioritized working memory predict item accessibility on a trial-by-trial basis

**Figure 5.**
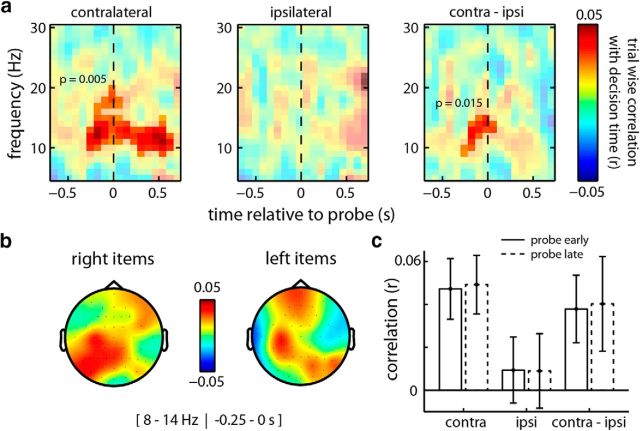
Trialwise correlation between preprobe contralateral α power and working memory access times. ***a***, Time-frequency map of the trialwise correlation between preprobe and postprobe power and decision times, separately for channels contralateral (leftmost) and ipsilateral (middle) to the location of the probed item at encoding, as well as for the difference in correlation between the contralateral and ipsilateral channels (rightmost plot). Transparency masks highlight the significant time-frequency cluster (see Materials and Methods). Only valid trials (in which the sides of the expected item and the probed item were the same) were included in the analysis. ***b***, Topography plots of the trialwise correlation between preprobe α power and decision times, separately for probed items that were on the right and on the left at encoding. ***c***, Bar graph represents the trialwise correlations of interest between preprobe α power and decision times, separately for early and late probes.

Finally, we investigated whether the identified index of prioritized working memory states also predicts working memory performance on a trial-by-trial basis. For this analysis, we centered our window of interest on the probe onset and focused exclusively on valid trials. Only in these trials are the expected and the probed item the same item, such that contralateral and ipsilateral (again, relative to the side of the item at encoding) can be defined relative to both preprobe and postprobe time-points. To increase sensitivity, we initially collapsed across early and late probes (we separate them again later). Whereas no significant trialwise correlation was observed between time- and frequency-resolved power and reproduction errors, we found significant trialwise correlations with our decision time variable, as depicted in Figure 5.

In contralateral channels, we observed a significant time-frequency cluster (cluster *p* = 0.005) that again centered in the α band. This cluster started before probe onset and extended well into the postprobe decision window (Fig. 5*a*, left). This positive cluster implies that trials with higher α power were associated with slower decisions, or, equivalently, that lower α power was associated with faster decisions. Importantly, this correlation with behavioral performance was absent for channels ipsilateral to the side of the item during encoding (Fig. 5*a*, middle). Moreover, when contrasting contralateral and ipsilateral contributions directly (Fig. 5*a*, right), a significant cluster (cluster *p* = 0.015) survived that was specific to the 250 ms interval immediately preceding the probe. It is again critical to note that probes were always presented centrally, such that any lateralized effect must be attributed to retention-related activity.

The topographies of this lateralized preprobe effect supported a predominantly contralateral contribution. As depicted in Figure 5*b*, for items that were presented on the right during encoding, primarily the left (contralateral) posterior sites revealed a positive correlation between α power and decision time. Likewise, when considering items that were presented on the left during encoding, this effect appears shifted to the right posterior sites. The latter topography additionally highlights a left medial contribution that appears present for both left and right items. Because participants always responded with their right hand, it is conceivable that fluctuations in α power in the left motor cortex also impacted on decision time.

Finally, we assessed whether preprobe α power was predictive of performance in both early and late probe trials. Interestingly, as depicted in Figure 5*c*, trialwise correlations and their contralateral specificity were highly similar for early and late probes (despite the observation that the expectation-related α modulation was only clearly visible in the late interval).

## Discussion

We investigated whether and how temporal expectations guide the dynamic prioritization of representations held in visual working memory, and made two key advances. First, we demonstrate that internally guided temporal expectations can have a profound influence on working memory performance, revealing faster access as well as more accurate reproduction for mnemonic items that are probed at expected times. Second, we show that this attentional prioritization in working memory is associated with the spatially and temporally specific attenuation of posterior α oscillations that, moreover, predicts working memory access times on a trial-by-trial basis. We discuss each of these points in turn.

### Attentional dynamics in working memory

Despite the notion that working memory is typically conceived of as a highly dynamic store that serves the active retention of the currently most relevant information, recent tasks investigating its retention mechanisms tend to be relatively “static” settings, requiring participants to retain a set number of items throughout the same delay interval. Our results show that, even when a fixed number of items are retained in working memory, individual items can be dynamically prioritized at their most relevant times.

Initial support for a more dynamic view of working memory representations comes from studies demonstrating that attentional cues presented during a working memory delay (retro-cues) can strongly facilitate performance for cued items (Griffin and Nobre, 2003; Landman et al., 2003; for review, see Souza and Oberauer, 2016). To date, however, this work has focused predominantly on spatial and/or object-based attentional influences. Complementing this work, the current results show that temporal attention also continues to operate during working memory, thereby also complementing a large body of work demonstrating profound influences of temporal attention in the perceptual domain (Coull and Nobre, 1998; Ghose and Maunsell, 2002; Nobre et al., 2007; Rohenkohl et al., 2014). In particular, we show that attentional prioritization in working memory can be highly dynamic (i.e., reversible) and temporally precise. In addition, the current work reveals that such prioritization can be internally guided (i.e., can proceed without retro-cues), thereby providing a more ecological demonstration of attentional prioritization in working memory.

One particularly interesting aspect of our behavioral data was the dynamic nature by which previously unprioritized items (i.e., items expected late) could again gain priority once the early interval had passed (Lewis-Peacock et al., 2012; LaRocque et al., 2013; see also Rerko and Oberauer, 2013; Zokaei et al., 2014). These findings suggest that the initial prioritization of one item (in the early interval) does not necessarily impair the representational fidelity of the other (unprioritized) items, at least when the total number of items in working memory does not exceed one's capacity (Astle et al., 2012). Rather, these items can be dynamically put back into the focus of attention to benefit subsequent performance.

Considering the mechanisms by which temporal expectations influence working memory performance, it is relevant to note that this influence was most pronounced for decision times. This suggests that prioritized (expected) items are in a more accessible state such that, when probed, their representations can be accessed and acted upon faster. Conversely, when probed about a currently unprioritized (unexpected) item, attention may first need to shift back to this item, rendering a longer interval before a decision is made. Our data suggest that such refocusing of attention (as prompted by an invalid probe) takes ∼100 ms, which is in line with object-switch costs observed when switching between two items (Oberauer, 2006). Possibly, this influence of temporal expectations on item accessibility also contributes to the observed influence on reproduction accuracy. Provided that participants may automatically feel pressured to complete their reproduction response as quickly as possible, longer access times may result in less precise reproduction reports. Alternatively, the observed accuracy effects may also be due to other mechanisms that have been proposed to underlie attentional prioritization in working memory, such as increased protection from interference by the probe (Makovski et al., 2008; Souza and Oberauer, 2016).

### α oscillations in visual working memory

Cortical α oscillations are a reliable marker of the engagement of the underlying neuronal populations, with lower amplitudes reflecting higher engagement (Klimesch et al., 2007; Jensen and Mazaheri, 2010; Foxe and Snyder, 2011). Indeed, cortical α power is negatively correlated with neuronal spiking activity (Haegens et al., 2011), BOLD signals (e.g., Goldman et al., 2002), and performance in perceptual detection tasks (e.g., van Dijk et al., 2008; van Ede et al., 2012). Moreover, the amplitude of these oscillations can be dynamically regulated by voluntary attention to serve the prioritization of relevant over irrelevant (anticipated) sensory information (Foxe et al., 1998; Worden et al., 2000; Thut et al., 2006; van Ede et al., 2012). Our data suggest that attenuated α oscillations over contralateral posterior sites (putatively, visual cortices) also support the prioritization of mnemonic representations held in visual working memory.

Previous studies have already implicated α oscillations in working memory retention. An influential paper by Jensen et al. (2002) showed that the amplitude of posterior α oscillations increases with working memory load. As their paradigm required verbal rehearsal of the encoded items, it was suggested that this increase may reflect the disengagement of the task-irrelevant visual areas (see also Klimesch et al., 2007; Roux and Uhlhaas, 2014). Indeed, in sensory areas relevant to the retained perceptual information, α oscillations have since been shown to be attenuated during working memory (e.g., Sauseng et al., 2009; Spitzer and Blankenburg, 2012). Based on the latter work, it has further been hypothesized that the degree of α attenuation during working memory retention may reflect the degree to which the mnemonic representations (which are retained by the underlying neuronal populations) are prioritized (Myers et al., 2014; Poch et al., 2014; Wallis et al., 2015; Mok et al., 2016).

The current results extend previous studies relating attenuated α oscillations to prioritization in visual working memory in two key ways. First, in previous studies, lateralized probe anticipation and retro-cue processing may have also contributed to the lateralized α modulations during retention. In the current study, the probe was always presented centrally and attentional shifts were internally guided. Therefore, the lateralized α modulation observed here must be attributed to dynamic processes during the working memory retention period that occurs relative to the location of the mnemonic item at encoding. Second, we reveal a trialwise association between the degree to which contralateral α oscillations are attenuated during the retention interval and the speed with which mnemonic representations can be accessed for report. To our knowledge, such a trialwise correlation with visual working memory performance has previously only been reported for α oscillations before encoding (Myers et al., 2014).

Interestingly, in the context of a somatosensory working memory task, Haegens et al. (2010) previously reported that optimal working memory performance was associated with enhanced α oscillations ipsilateral to the location of the mnemonic item at encoding (Haegens et al., 2010). In contrast, we only found evidence that optimal working memory performance was associated with attenuated contralateral α oscillations. Whether this discrepancy may be attributed to a sensory modality difference, or to other differences between the used working memory tasks, remains to be resolved.

### Absence of an attentional α modulation during working memory prioritization in the early interval

Despite the fact that the behavioral prioritization effects were largely symmetrical between the early and the late intervals, the attentional α modulation was only significant in the late interval. We consider three possible reasons that may underlie this apparent discrepancy. First, in our design, the probability of being probed for either item at some point in the trial is 50/50 from the outset but changes to 80/20 after the first interval has passed. This “imbalance” may account for larger (neuronal) attentional modulations in the late interval. Second, because the early interval immediately follows encoding, there are likely many additional computations taking place in visual cortex that also impact on α oscillations, thereby possibly obscuring their attentional modulation. For example, bilateral visual stimulation will attenuate bilateral α oscillations and may abolish attentional modulations by virtue of a “floor effect” (see also van Ede et al., 2014). Third, only in the late interval may time estimation have become so imprecise that it is beneficial to upregulate the relevant mnemonic representation well in advance of the probe, as manifested in the sustained α modulation. Disentangling these possibilities remains an important target for future research.

Interestingly, although we could not confirm the presence of a clear attentional α modulation in the early interval, α power in this interval was still predictive of performance. This suggests that “spontaneous fluctuations” (i.e., fluctuations unrelated to the experimental manipulation) in α power also translate into fluctuations in the prioritization of mnemonic representations.

In conclusion, internally guided temporal expectations can dynamically guide which item in visual working memory is currently prioritized. This influence is most prominent on the accessibility of mnemonic representations and is at least partly supported by the temporally specific attenuation of contralateral α oscillations that is relevant for performance.
